# Comparative Floral Development and Anatomy Reveal Distinct Origins of the Gynophore in Meso-Papilionoideae

**DOI:** 10.3390/plants15030426

**Published:** 2026-01-30

**Authors:** Cinthia Gracielly Rodrigues, Sueli Maria Gomes

**Affiliations:** 1 Instituto Federal de Educação Ciência e Tecnologia do Norte de Minas Gerais, Instituto Federal do Norte de Minas, Arinos 38680-000, Brazil; 2 Programa de Pós-Graduação em Botânica, Universidade de Brasília, Brasília 70910-900, Brazil; suelimariagomes@gmail.com

**Keywords:** *Andira vermifuga*, common primordia, dalbergioid clade, Fabaceae, genistoid clade, *Leptolobium brachystachyum*, *Luetzelburgia auriculata*, ontogeny, secretory structures, *Vatairea macrocarpa*

## Abstract

The Meso-Papilionoideae clade comprises most papilionoid legumes and includes small clades with heterogeneous floral morphologies. Some species have a sessile ovary, while in others the gynoecium is elevated by a stalk called a stipe or gynophore. This study provides a qualitative and comparative morphological analysis of meso-papilionoid flowers, focusing on the anatomy, vascularization, and development of the ovary and gynophore. The objective is to unravel the ontogenic origin and anatomical nature of the gynophore in meso-papilionoid flowers. Floral buds at different developmental stages of seven meso-papilionoid species were examined using scanning electron microscopy and sectioned transversely and longitudinally for analysis under optical microscopy. The morphological variations in the examined flowers may represent evolutionary adaptations associated with their respective pollination syndromes. Ovary development follows a certain pattern among legumes, with limited variations, including the formation of a basal pedestal beneath the carpel suture in species bearing a gynophore. The gynophore is anatomically distinct from the ovary, exhibiting a stem-like nature, originating from the activity of an intercalary meristem on the basal pedestal of the ovary or receptacle. This qualitative anatomical approach represents a first step toward the homologation of gynophore types in Fabaceae, providing a basis for future quantitative and phylogenetic analyses.

## 1. Introduction

**Figure 1:**
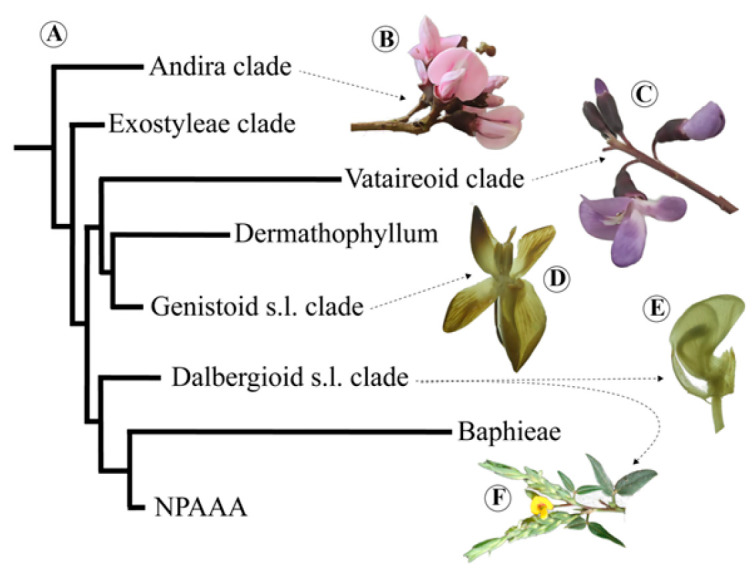
Phylogenetic tree of Meso-Papilionoideae with flowers of species investigated in this study. (**A**) Phylogenetic tree of Meso-Papilionoideae (50 kb Inversion Clade), obtained and modified from Choi et al. [3]. (**B**) *Andira vermifuga* (Mart.) Benth. flowers, (**C**) *Vatairea macrocarpa* (Benth.) Ducke flowers, and (**D**) photograph under stereomicroscope of open pre-anthetic flower bud of *Crotalaria paulina* Schrank showing a pair of callosities at the base of the standard blade. (**E**) Photograph under stereomicroscope of part of the flower of *Arachis hypogaea* L. (**F**) Apex of the spike-shaped inflorescence of *Zornia latifolia* Sm.

Meso-Papilionoideae is the name proposed by Wojciechowski [1] for a large monophyletic group of papilionoids (Fabaceae), marked by the inversion of a 50 kb region in the plastid genome, informally called the “50 kb Inversion Clade” [2]. The Meso-Papilionoideae clade (Figure 1A) comprises 98% of Papilionoideae and includes the clades Genistoid s.l., Dalbergioid s.l. and species-rich Baphieae + NPAAA (Non-Protein Amino Acid-Accumulating) clades, the small clades Andira, Exostyleae, and Vataireoid, as well as the phylogenetically unresolved genera *Dermatophyllum* Scheele and the African *Amphimas* Pierre ex Harms [3]. The NPAAA is the most diverse clade and includes the most economically important papilionoid legumes.

In this study, flowers from representative species of some subclades were analyzed. *Andira vermifuga* (Mart.) Benth. belongs to the Andira clade, which predominantly comprises species from the neotropical rainforest [4]. This clade exhibits a diversity of floral traits, such as actinomorphic flowers with free stamens (*Aldina* Endl.) and papilionaceous flowers (*Andira* Lam. and *Hymenolobium* Benth.) [3]. The Vataireoid clade, exclusively neotropical, is represented here by *Luetzelburgia auriculata* (Allemão) Ducke and *Vatairea macrocarpa* (Benth.) Ducke [5]. Additionally, two representatives of the genistoid clade were included: *Leptolobium brachystachyum* (Benth.) Sch.Rodr. & A.M.G.Azevedo, an endemic species from the rocky Brazilian cerrado, which has radially symmetrical flowers, and *Crotalaria paulina* Schrank with papilionaceous flowers. The Leptolobieae clade includes trees mostly from tropical rainforests and savannas of South America and one species endemic to Central America [6,7]. In contrast, the Crotalarieae clade, a subclade of the Core Genistoids, is mostly endemic to Africa and by far considered the largest subclade within the Genistoids s.l. [6,8]. Finally, the papilionaceous flowers of the dalbergioid species *Zornia latifolia* Sm. and *Arachis hypogaea* L. were also included in this study. The genus *Arachis* L. belongs to the Pterocarpus clade and is naturally confined to South America, with the majority of its species native to Brazil [9,10]. *Arachis hypogaea* is particularly relevant as it is cultivated as an oilseed and a major food source. The genus *Zornia* J.F.Gmel. is a member of the informal Adesmia clade of the Dalbergioid group, being most closely related to the predominantly South American genera *Poiretia* Vent. and *Amicia* Kunth [11].

The floral morphological diversity of species representing these clades was explored in this work with a focus on the basal stalk of the ovary. A considerable number of papilionoid legumes have a stalk that elevates the gynoecium, called a stipe or gynophore [12,13]. Fabaceae flowers show variation in gynophore morphology, which may appear as a free structure or attached to the hypanthium wall, elongated or short, hairy or glabrous, and surrounded or not by an annular nectary [14,15,16,17]. In this study, two species have a sessile ovary (*Z. latifolia* and *C. paulina*), one has a short gynophore (*L. auriculata*), three have a long gynophore (*A. vermifuga*, *V. macrocarpa*, and *L. brachystachyum*), and one has a special feature, which is the elongation of the very long gynophore after pollination (*A. hypogaea*). The gynophore deserves to be better investigated in morphoanatomical and ontogenetic studies for the appropriate use of its nomenclature. The term gynophore refers to a structure distinct from the carpel, with an anatomical nature and vascularization similar to the stem and originating through intercalary growth from the receptacle [18,19]. The use of the term gynophore in *Arachis* is controversial. According to Smith [20], the fruit stalk of *Arachis* would have its origin in the elongation of the ovary itself. Therefore, this structure would not be a gynophore (of stem origin), but an elongated ovary. However, the anatomical stem nature of the *Arachis* gynophore is undeniable [21].

Ontogenetic studies in legumes suggest the origin of the gynophore from the basal pedestal [22,23,24]. This term, basal pedestal, was used by Tucker [25] to designate the unplicated region located below the carpel suture in the early stages of ovary development in detarioids. A more recent study showed that the gynophore of *Bowdichia virgilioides* Kunth has a stem-like nature and originates from the floral receptacle [26].

Therefore, here we propose a qualitative investigation of the general floral morphology of meso-papilionoid species, as well as a specific analysis of the anatomy and vascularization of the ovary and gynophore. Our objective is to unravel the ontogenic origin and anatomical nature of the gynophore of these species and access synapomorphic characters between genera of the same clade. This qualitative approach seeks to provide support for future quantitative and phylogenetic analyses, contributing to the understanding of floral evolution in Fabaceae.

## 2. Results

### 2.1. Organography

*Andira vermifuga* exhibits terminal and axillary panicles comprising about 12 flowers on a side branch. Floral buds are fusiform in shape, covered with pale brown trichomes. Flowers (Figure 1B) are 2 cm long, and the calyx is brown to purple-brown, five-lobed, with pale brown trichomes. The corolla comprises five free, pink, and clawed petals. The standard petal is emarginate, has violet streaks, and a white spot in the middle. The androecium is composed of 10 heterogeneous stamens, globose and dorsifixed anthers, and longitudinal dehiscence. The carpel, up to 15 mm long, has an unilocular superior ovary, sparsely hairy on the lower surface. The ovary is elevated by the gynophore up to 7 mm long. The style is slightly curved, and the stigma is cristate.

*Arachis hypogaea* has axillary, spike-like inflorescences. At each node of the minute branches of the inflorescence, a lower bract is born, which subtends the floral bud born in its axil, as well as all the younger floral buds born in the nodes above. In addition to the lower bract, each floral bud is individually subtended by two partially fused bracteoles. Usually, only one flower of an inflorescence reaches anthesis on a given day. Flowers (Figure 1E) are sessile. The calyx is green, five-toothed, with four teeth united on the adaxial side, opposite the standard petal, and one abaxial sepal, opposite the lower bract. Sepals have long trichomes similar to those on the bracts. The corolla comprises five yellow petals. The standard petal is emarginate, and its base is broadly fused with the base of the staminal column. The wings and keel petals are attached by means of claws, the bases of which are adnate to the base of the staminal column. The keel petals cover the staminal column and are fused along their dorsal edges to the apex, but open ventrally at the base. The androecium consists of eight functional stamens and two staminodes. One staminode is antepetalous, opposite the standard; the other is antesepalous, alternating with the standard and wing petals. The filaments fuse for at least two-thirds of their length. The functional stamens have dimorphic anthers (four introrse and oblong anthers alternately with four dorsifixed and globose anthers). The carpel, up to 45 mm long, has an unilocular ovary up to 1.5 mm long. The style is filiform, greatly elongated, slightly curved at the apex, and is clothed with a series of trichomes below the stigma on the surface facing the standard petal. At the end of the staminal column, the style is reflexed parallel with the filaments but extends beyond the anthers to its terminus in the beak of the keel. The stigma is clavate.

*Crotalaria paulina* exhibits terminal racemes with about 30 flowers arranged on the branch. Flower buds are falcate and green. Flowers, up to 3.5 cm long, are pedicellate. The calyx consists of two fused sepals, opposite the standard, and three fused sepals, opposite the keel petals. The corolla comprises five yellow petals (Figure 1D). Keel petals are fused. The androecium is composed of 10 heterodynamous stamens. The staminal tube is open on one side of the vexillary stamen. The anthers are dimorphic (five oblong and basifixed anthers alternate with five globose and dorsifixed anthers). Approximately one day after anthesis, the short, dorsifixed anther filaments elongate and extend past the long, basifixed ones. The carpel, up to 8 mm long, has a unilocular, glabrous, sessile ovary. The style is erect, and the stigma is cristate.

*Leptolobium brachystachyum* has terminal panicles. Flower buds are cuneate and pale brown. Flowers, up to 11 mm long, are pedicellate. The calyx is glabrescent, five-laciniate, with valvate aestivation. The corolla comprises five free, white petals, similar in size and shape. The androecium is composed of 10 free, heterodynamous stamens, with globose and dorsifixed anthers. The carpel, up to 9 mm long, has a tomentose ovary supported by a glabrous gynophore up to 3 mm long. The style is curved, and the stigma is punctiform.

*Luetzelburgia auriculata* exhibits terminal panicles with flowers up to 16 mm long. Flower buds are elliptical in shape. The calyx is tomentose, five-toothed. The corolla is nearly papilionaceous, with five free, white petals, auriculate, crinkled, and with trichomes on the abaxial surface. The middle portion of the standard petal is thick and fleshy, with a dark purple stain. Lateral (wing-like) and abaxial (keel-like) petals are undifferentiated in shape but slightly differentiated in size and overlapping. The androecium is composed of nine heterodynamous stamens (the vexillary stamen is suppressed), basally connate filaments, with globose, dorsifixed anthers. The carpel, up to 15 mm long, has a tomentose ovary, laterally compressed, raised by the gynophore, and up to 2 mm long. The style is very slightly curved at the apex, and the stigma is punctiform.

*Vatairea macrocarpa* has terminal panicles with pedicellate flowers up to 25 mm long (Figure 1C). Flower buds are fusiform in shape. The calyx is tomentose, campanulate, five-toothed, and vinaceous. The corolla is papilionaceous, with five free purple petals. The standard petal is emarginate and auriculate. The central portion of the standard petal has a white stain, and the base is thick and fleshy. The wing and keel petals are auriculate. Lamellate sculptures are present externally on the wing petals. The androecium is monadelphous, composed of ten heterodynamous stamens with oblong, dorsifixed anthers. Filaments are fused until about three-quarters of their length. The staminal tube is open on one side of the vexillary stamen. The carpel, up to 23 mm long, is laterally compressed, densely tomentose, unilocular, with one ovule, and elevated by the pilose gynophore up to 8 mm long. The style is slightly curved, and the stigma is punctiform.

*Zornia latifolia* has axillary, spike-like inflorescences with sessile flowers up to 9.5 mm long (Figure 1F). The calyx is green, five-toothed, with trichomes on the edges and along the rib located in the middle of the three abaxially fused sepals. The corolla comprises five yellow petals. The standard petal has red streaks in its central portion and a small opening in the lower central portion. The wing petals have claws and lunate sculpturing. Keel petals cover the staminal column and are fused along its dorsal and ventral edges, but open dorsoventrally at the base. The androecium consists of ten stamens heterodynamous with filaments fused until at least three-quarters of their length. The anthers are dimorphic (five basifixed and oblong anthers alternately with five dorsifixed and globose anthers). The carpel, up to 12 mm long, has a unilocular, sessile ovary up to 6 mm long. The style is erect or slightly curved, and the stigma is cristate.

The floral diagrams, floral formulae, and morphological characters of the species examined are summarized in Table 1.

### 2.2. Carpel Ontogeny

The results of carpel ontogeny are presented in a comparative manner between species of the same clade. These results are also summarized in Table 1.

**Figure 2:**
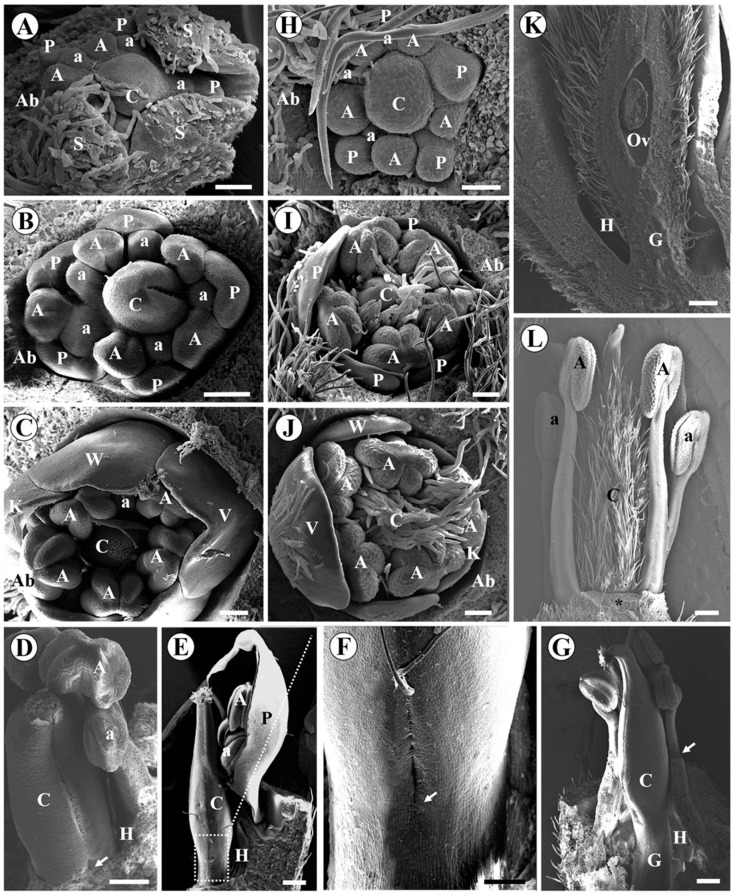
Scanning electron micrographs of floral development of *Andira vermifuga* (**A**–**G**) and *Luetzelburgia auriculata* (**H**–**L**). Bracteoles and some sepals removed in (**A**). Bracteoles and all sepals removed in (**B**–**L**). *A. vermifuga*: (**A**) Polar view of flower bud showing all floral organs initiated; (**B**) polar view showing formation of the carpel cleft, the beginning of petal differentiation, and the formation of the thecae; (**C**) polar view showing carpel apex with a papillate surface, differentiation of corolla petals, and formation of microsporangia; (**D**) lateral view of the carpel showing carpel cleft closure, the basal pedestal (arrow), and the beginning of hypanthium formation; (**E**) long trichomes on the stigmatic surface; elongation of the style, hypanthium, and gynophore; (**F**) enlarged view of the region highlighted in (**E**) showing the end point of carpel closure (arrow); (**G**) the style becomes curved, the gynophore grows larger, and the filaments fuse by intercalary growth (arrow). *L. luetzelburgia:* (**H**) polar view of flower bud showing all floral organs initiated; (**I**) polar view of the flower bud showing the carpel with numerous trichomes, the carpel slit and the formed microsporangia, and the differentiation of the petals from the corolla; (**J**) trichomes completely cover the carpel and are present on the adaxial surface of the petals. (**K**) Floral bud longitudinally sectioned, showing unilocular ovary with a single ovule, short hypanthium, and a gynophore. (**L**) Stigma and style formed; absence of the vexillary stamen (asterisk). Abbreviations: A = antesepalous stamen; a = antepetalous stamen; Ab = abaxial side; C = carpel; G = Gynophore; H = Hypanthium; K = keel petal; Ov = ovule; P = petal; S = sepal; V = vexillum petal; W = wing petal. Scale bar = 50 µm in (**A**,**H**); 100 µm in (**B**–**D**,**F**,**I**,**J**); 300 µm in (**E**,**G**,**K**,**L**).

In *A. vermifuga* and *L. auriculata*, the carpel probably emerges together with the antesepalous stamens. We estimated it due to the size and shape of the carpel primordium during the initiation of antepetalous stamens (Figure 2A,H). In *A. vermifuga*, it is possible to visualize the carpel cleft at the moment when the petal primordia acquire a dorsiventral shape, and the anthers become adaxially bilobed (formation of thecae) (Figure 2B). Subsequently, the formation of the stigma occurs concomitantly with the differentiation of the microsporangia and differentiation of the petals into one vexillum, two wings, and two keels (Figure 2C). In *L. auriculata*, carpel differentiation is later. The carpel cleft is visible along with the formation of microsporangia and corolla differentiation (Figure 2I). Furthermore, the carpel and the abaxial surface of the petals present numerous trichomes (Figure 2J).

In *A. vermifuga*, the carpel cleft closes, and the apical portion of the carpel curves slightly. The region below the common bases of sepals, petals, and stamens expands to form the hypanthium. The carpel suture does not extend to the base of the carpel. There is a short basal pedestal in this region (Figure 2D). The short basal pedestal lengthens in the final stages to form the gynophore (Figure 2E,F). The style becomes curved, and the filaments fuse for at least half of their length by intercalary growth (Figure 2G), except the vexillary stamen, forming the diadelphous androecium. In *L. auriculata*, the short gynophore and hypanthium elongate in the final stages (Figure 2K). The style and stigma form, the filaments fuse only at the base, and the vexillary stamen is absent (Figure 2L).

**Figure 3:**
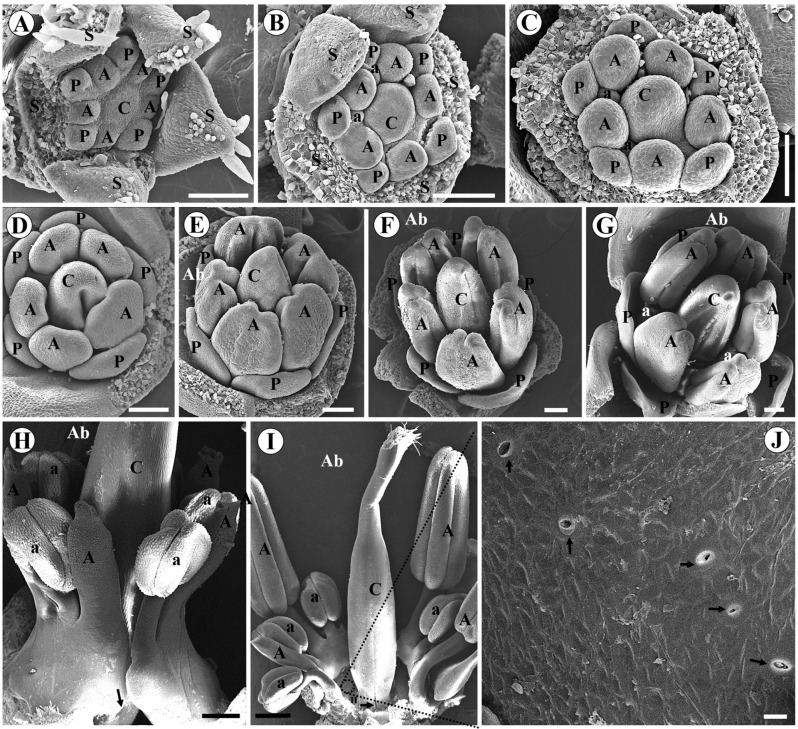
Floral development in *Crotalaria paulina* (scanning electron micrographs). Bracteoles were removed in all figures. The abaxial side is at the top in (**A**–**D**) and is labeled in (**E**–**I**). (**A**) Carpel initiation concurrently with the antesepalous stamens; one sepal was removed; (**B**) initiation of the antepetalous stamens on the abaxial side; (**C**) growth of carpel margins to initiate carpel cleft formation, before initiation of all antepetalous stamens; (**D**) the stamens begin to differentiate, forming the thecae, the carpel cleft deepens, and the petal margins grow. (**E**) the carpel grows, its apex tapers, and microsporangia are formed; (**F**) carpel cleft closure and longitudinal elongation of the anthers of the antesepalous stamens; (**G**) there is a small deepening at the apex and the ventral surface of the carpel before the style elongation; (**H**) the antepetalous and antesepalous stamens are reoriented into a single whorl and their filaments become fused into an open tube. The two stamens, opposite the standard and the carpel suture, do not merge but overlap, forming a nectar window (arrow) beneath them. (**I**) Elongated style, long trichomes on the surface of the stigma, the carpel closure extends to the base of the carpel (arrow), and short hypanthium. Note a dilated region in the hypanthium, around the base of the ovary. (**J**) Enlarged view of figure I (circle), showing putative modified stomata (arrow) on the dilated region of the hypanthium wall. Abbreviations: A = antesepalous stamen, a = antepetalous stamen, Ab = Abaxial side, C = carpel, H = Hypanthium, P = petal, S = sepal. Scale bars: (**J**) = 10 μm; (**A**–**G**) = 100 μm; (**H**) = 300 μm, (**I**) = 500 μm.

**Figure 4:**
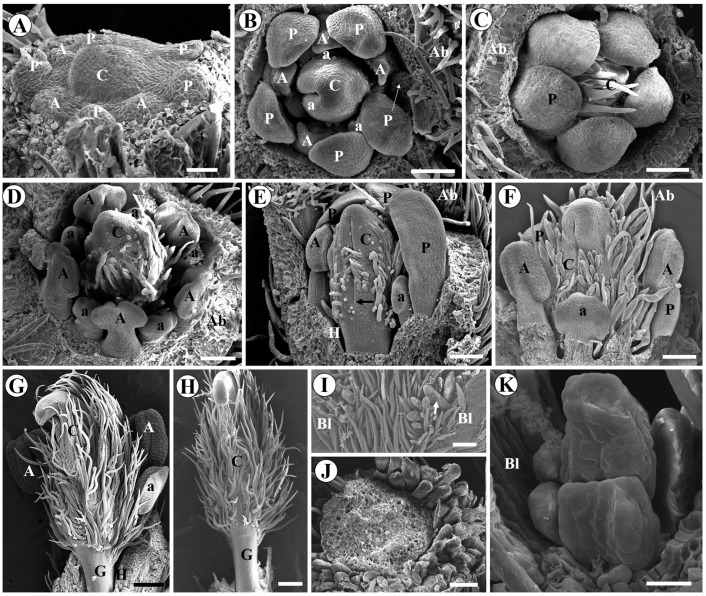
Floral development in *Leptolobium brachystachyum* (scanning electron micrographs). Bracteoles were removed in (**A**–**H**). The abaxial side is at the top in (**A**) and is labeled in the other figures. (**A**) Initiation of the carpel together with the antesepalous stamens; (**B**) the carpel cleft is evident and trichomes emerge on the dorsal surface of the carpel; (**C**) the trichomes on the dorsal surface of the carpel grow and the petals have a shell shape; (**D**) formation of the microsporangia; (**E**) trichomes emerge on the ventral surface of the carpel; the carpel cleft closure does not extend to the base of the carpel (arrow); beginning of hypanthium formation; (**F**) curvature of the apex of the carpel to form the stigma and numerous trichomes covering the ventral and dorsal surfaces of the carpel; (**G**) elongation of the hypanthium, gynophore and style; papillae emerge on the stigma; (**H**) hairy carpel and glabrous gynophore; (**I**) colleter-like glands (arrow) in the axil of bracteoles; (**J**) colleter-like glands around the flower bud; all the organs of the flower bud were removed; (**K**) enlarged view of the colleter-like glands. Abbreviations: A = antesepalous stamen, a = antepetalous stamen, Ab = Abaxial side, Bl = Bracteole, C = carpel, G = Gynophore, H = Hypanthium, P = petal. Scale bars: (**A**,**K**) = 20 μm; (**B**–**F**,**I**,**J**) = 100 μm; (**G**,**H**) = 200 μm.

In *C. paulina* and *L. brachystachyum*, carpel initiation also occurs together with the antesepalous stamens (Figure 3A and Figure 4A). In *C. paulina*, the antepetalous stamens begin abaxially, while the carpel and other organs increase in size (Figure 3B). The edges of the carpel expand laterally to initiate the formation of the carpel cleft (Figure 3C). In both species, the carpel cleft becomes evident at the same time as the formation of the thecae occurs and the corolla begins to differentiate (Figure 3D and Figure 4B). In *L. brachystachyum*, trichomes emerge on the dorsal surface of the carpel (Figure 4B). The petals acquire a shell shape, and the trichomes on the carpel grow (Figure 4C). In both species, the carpel grows, its apex thins, and differentiation of microsporangia occurs (Figure 3E and Figure 4D). In *C. paulina*, the anthers of the antesepalous stamens grow longitudinally, and the carpel cleft closes (Figure 3F). A small depression forms at the apex and ventral surface of the carpel (Figure 3G). In *L. brachystachyum*, trichomes emerge on the ventral surface of the carpel, and the hypanthium begins to lengthen. The carpel closure does not extend to the base of the carpel, forming a small, glabrous basal pedestal (Figure 4E).

In *C. paulina*, the antesepalous and antepetalous stamens reorganize into a single whorl, and their filaments fuse until about a third of their length, forming the staminal tube. The tube has an opening between the two stamens opposite the standard, whose filaments do not merge, forming a nectar window (Figure 3H). The style lengthens and the cristate stigma forms. The carpel closure extends to the base of the carpel (Figure 3I). At the wall of the very short hypanthium there is a thick region, below the insertion of the filaments and around the base of the carpel, with several putative modified stomata (Figure 3I,J).

In *L. brachystachyum*, the apex of the carpel becomes curved, and the stigma forms (Figure 4F). The style, hypanthium, and gynophore lengthen, and the surface of the stigma becomes papillate (Figure 4G,H). There are colleter-like glands around the base of the flower bud (Figure 4J) and in the axils of the bracteoles (Figure 4I,K).

**Figure 5:**
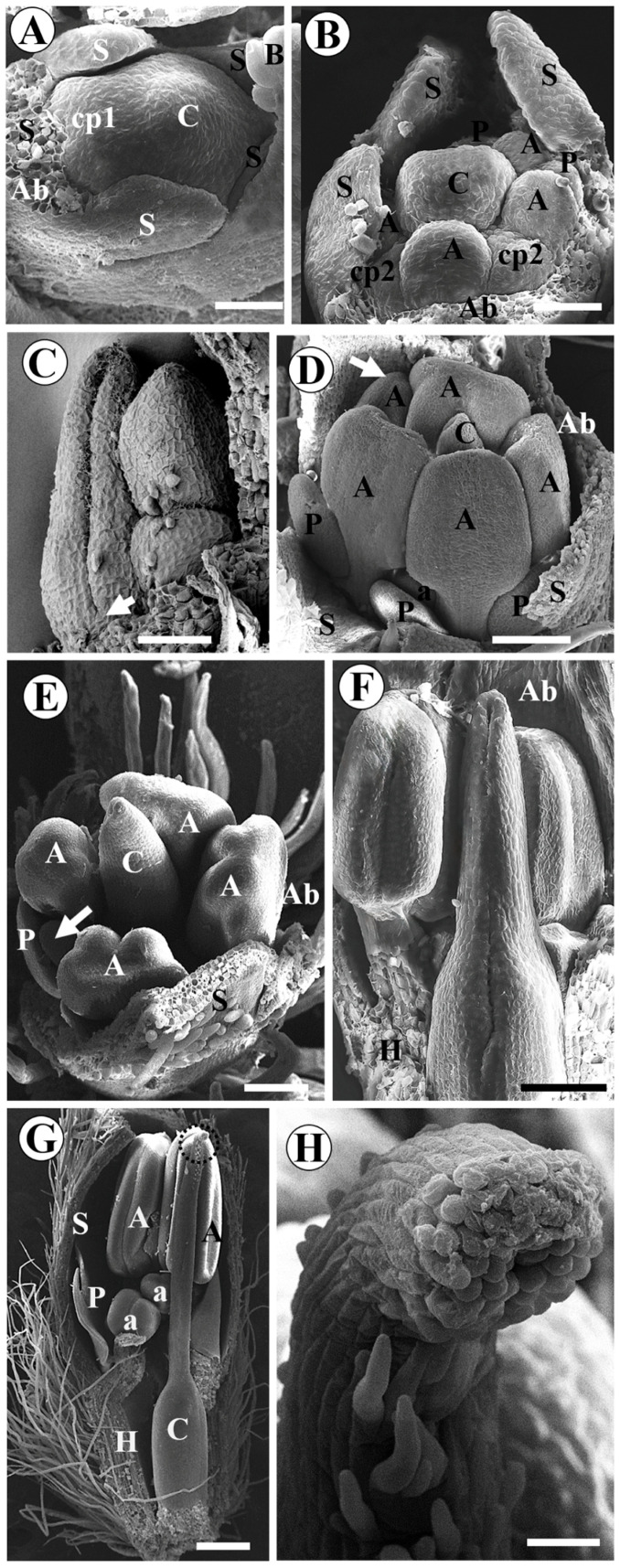
Floral development in *Arachis hypogaea* (scanning electron micrographs). Bracts have been partially removed in (**A**) and completely removed in the other figures. The abaxial side is labeled in all figures. (**A**) Initiation of the carpel concurrently with common petal-stamen primordia; (**B**) two secondary common petal-stamen primordia form abaxially, while the carpel grows; (**C**) the carpel cleft closure does not extend to the base of the gynoecium (arrow); (**D**) one of the antesepalous stamens near the standard petal develops less than the others (arrow); (**E**) the antesepalous stamen near the standard petal cease its development (arrow), while the apex of the carpel tapers; (**F**) elongation of the style and hypanthium; (**G**) papillae emerge on the surface of the stigma and just below it; (**H**) enlarged view of (**G**) (circle), showing the stigma with a papillate surface and trichomes in the style region close to the stigma. Abbreviations: A = antesepalous stamen, a = antepetalous stamen, Ab = abaxial side, B = bract, C = carpel, cp1 = primary common primordia, cp2 = secondary common primordia, H = Hypanthium, P = petal, S = sepal. Scale bars: (**H**) = 20 μm; (**A**–**C**) = 50 μm; (**D**–**F**) = 100 μm; G = 300 μm.

**Figure 6:**
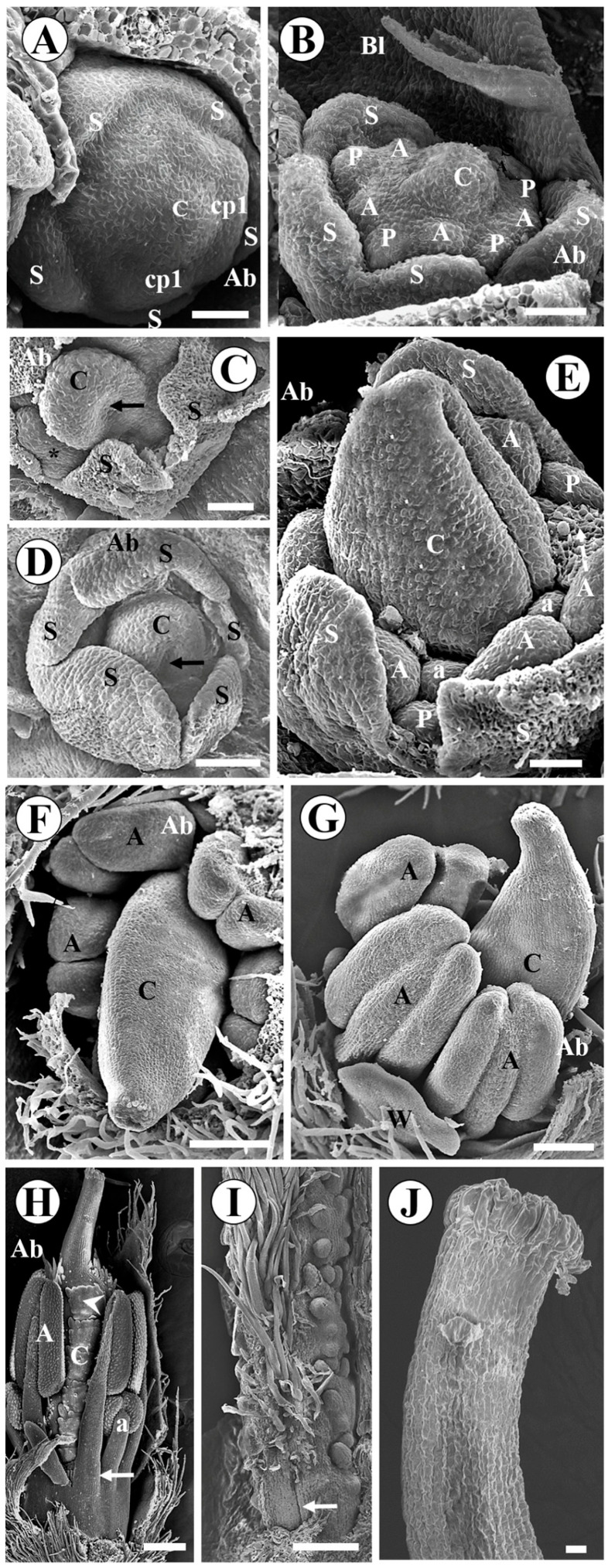
Floral development in *Zornia latifolia* (scanning electron micrographs). Bracteoles were completely removed in (**A**,**C**–**J**) and partially removed in (**B**). The abaxial side is labeled in all figures. (**A**) The carpel emerges along with two common stamen-petal primordia, abaxial and lateral; (**B**) the carpel grows while the antesepalous stamens and petals form from the common primordia; (**C**) beginning of the formation of the carpel cleft on the adaxial side (arrow). Note what appears to be a secondary common petal-stamen primordium that probably gives rise to the antepetalous stamens (asterisk); (**D**) the carpelar cleft is evident (arrow); (**E**) the carpel grows more than petals and stamens; (**F**) stigma formation; (**G**) style elongation and microsporangia formation; the anthers of the antesepalous stamens lengthen longitudinally, becoming oblong; (**H**) formation of articles (arrowhead) on the carpel; growth of trichomes on the ventral and dorsal surfaces of the carpel; basal fusion of filaments (arrow); (**I**) long and numerous trichomes on the ventral surface of the carpel, except in the basal region; the carpel suture extends to the base of the carpel; (**J**) expanded view of papillate stigma. Abbreviations: A = antesepalous stamen, a = antepetalous stamen, Ab = Abaxial side, Bl = Bracteole, C = carpel, cp1 = primary common primordia, P = petal, S = sepal, W = wing petal Scale bars: (**J**) = 10 μm; (**A**–**C**,**E**) = 30 μm; (**D**) = 50 μm; (**F**,**G**,**I**) = 100 μm; (**H**) = 200 μm.

In *Arachis hypogaea* and *Zornia latifolia*, the carpel emerges concurrently with the primary common petal-stamen primordia initially formed on the abaxial side (Figure 5A and Figure 6A). The antesepalous stamens and petals are formed from the primary common primordia (Figure 5B and Figure 6B). The antepetalous stamens are formed from secondary common primordia, which emerge later (Figure 5B and Figure 6C). In *Zornia latifolia*, the sepals begin to fuse when the carpel margins expand to form the carpel cleft (Figure 6C,D). The carpel grows longer than the stamens, which in turn grow longer than the petals (Figure 6E). The stigma undergoes differentiation and becomes papillate (Figure 6F). The anthers of the antesepalous stamens grow longitudinally, becoming oblong, and the style begins to lengthen (Figure 6G). The articles are formed in the ovary, the filaments fuse, and trichomes emerge late on the dorsal and ventral surfaces of the carpel (Figure 6H). The carpel cleft closure extends to the carpel base, which is glabrous (Figure 6I). In the final stages, numerous long papillae are evident on the stigmatic surface (Figure 6J).

In *A. hypogaea*, the carpel cleft does not extend to the base of the ovary. There is a very small base below the carpel suture (Figure 5C). The carpel grows together with the elongation of the filaments of the antesepalous stamens and the differentiation of the microsporangia (Figure 5D). The antesepalous stamen nearest the standard petal develops less than the other stamens and subsequently ceases its development. The apex of the carpel thins, initiating the formation of the style (Figure 5E). The style lengthens together with the hypanthium formation (Figure 5F). The style curves slightly at its apex near the stigma. The stigmatic surface becomes papillate. A series of trichomes is concentrated in the region just below the stigma (Figure 5G,H).

### 2.3. Anatomical Study of the Ovary and Gynophore

The anatomical characteristics are presented in a comparative manner. These results are also summarized in Table 1.

**Figure 7:**
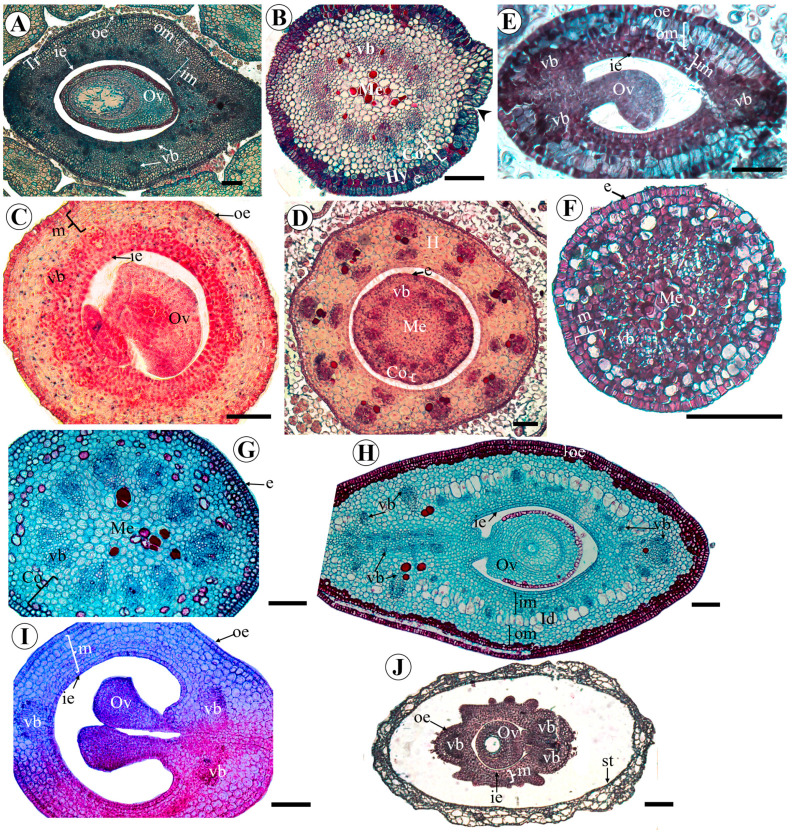
Transverse histological sections of the ovary and gynophore of *Vatairea macrocarpa* (**A**,**B**), *Arachis hypogaea* (**C**,**D**), *Leptolobium brachystachyum* (**E**,**F**), *Andira vermifuga* (**G**,**H**), *Crotalaria paulina* (**I**), and *Zornia latifolia* (**J**). (**A**) Hairy ovary of *V. macrocarpa*; (**B**) gynophore with trichomes and stoma (arrowhead) of *V. macrocarpa*; (**C**) ovary at the level of the ovule of *A. hypogaea*; (**D**) base of the ovary of *A. hypogaea* surrounded by hypanthium; (**E**) hairy ovary of *L. brachystachyum*; (**F**) gynophore of *L. brachystachyum;* (**G**) gynophore of *A. vermifuga*; (**H**) ovary of *A. vermifuga*; (**I**) ovary of *C. paulina*; (**J**) ovary and monadelphous androecium of *Z. latifolia*. Abbreviations: Co = cortex; e = epidermis; H = hypanthium; Hy = hypodermis; Id = idioblasts; ie = inner epidermis; im = inner mesophyll; m = mesophyll; Me = medule; oe = outer epidermis; om = outer mesophyll; Ov = ovule; st = staminal tube; Tr = trichomes; vb = vascular bundle. Scale bars: (**A**–**J**) = 100 μm.

In *A. vermifuga*, the outer epidermis of the ovary is glabrous and has two layers. The inner epidermis is uniseriate (Figure 7H). In *V. macrocarpa*, *A. hypogaea*, *L. brachystachyum*, *C. paulina*, and *Z. latifolia*, the outer and inner epidermis of the ovary are uniseriate (Figure 7A,C,E,I,J). The outer epidermis is glabrous in *A. hypogaea* (Figure 7C) and *C. paulina* (Figure 7I), but has numerous trichomes in *V. macrocarpa* (Figure 7A), *L. brachystachyum* (Figure 7E), and *Z. latifolia* (Figure 7J). In *Z. latifolia*, there are projections of the external epidermis, forming evaginations on the lateral walls of the ovary (Figure 7J). In *A. vermifuga* and *C. crotalaria*, the epidermis maintains some differentiated cells in the fundamental tissue, in the carpel cleft closure region (Figure 7H,I).

The ovarian mesophyll is heterogeneous in *V. macrocarpa*, *L. brachystachyum*, and *A. vermifuga* (Figure 7A,E,H). In *V. macrocarpa*, the outer mesophyll is composed of 4–5 layers of elongated parenchyma cells, and the inner mesophyll comprises 8–12 layers of flattened parenchyma cells (Figure 7A). In *L. brachystachyum*, the outer mesophyll is composed of two layers of elongated parenchyma cells, and the inner mesophyll comprises four layers of isodiametric parenchyma cells (Figure 7E). In *A. vermifuga*, the outer mesophyll is separated from the inner mesophyll by one to two layers of large cells (idioblasts). The outer mesophyll is made up of six to eight layers of partially isodiametric parenchyma cells, and the inner mesophyll is made up of six to eight layers of apparently smaller parenchyma cells, which become flatter closer to the inner epidermis (Figure 7H).

The ovarian mesophyll is homogeneous in *A. hypogaea*, *C. paulina*, and *Z. latifolia* (Figure 7C,I,J). In *A. hypogaea*, it is composed of 16 to 18 layers of isodiametric parenchyma cells (Figure 7C), while in *C. paulina*, it is composed of 8 layers of cells (Figure 7I). In *Z. latifolia*, the carpel mesophyll comprises six to nine layers of flattened parenchyma cells (Figure 7J).

In *L. brachystachyum* and *A. vermifuga*, the gynophore has a uniseriate and glabrous epidermis, a cortex composed of isodiametric parenchyma cells, and a vascular system organized in the form of a ring with vascular bundles, separated by parenchyma, around the parenchymal central medulla (Figure 7F,G). *V. macrocarpa* differs from these two species by presenting trichomes and stomata in the gynophore. In addition, the cortex has a hypodermis (Figure 7B).

**Figure 8:**
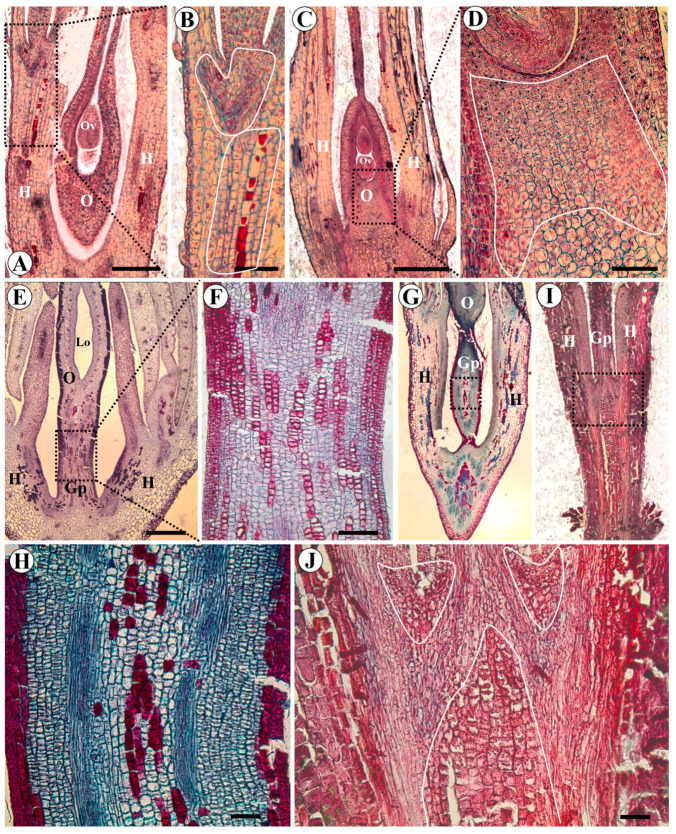
Longitudinal histological sections of flower buds showing the intercalary growth regions of the hypanthium and gynophore of *Arachis hypogaea* (**A**–**D**), *Andira vermifuga* (**E**,**F**), *Vatairea macrocarpa* (**G**,**H**), and *Leptolobium brachystachyum* (**I**,**J**). (**A**) General view of flower bud showing hypanthium of *A. hypogaea*; (**B**) enlarged view of (**A**) (rectangle), showing the intercalary meristem (highlighted area) responsible for the elongation of the hypanthium; (**C**) general view of the flower bud of *A. hypogaea* showing the ovary; (**D**) enlarged view of (**C**) (rectangle), showing intercalary meristem (highlighted area) responsible for gynophore elongation, located at the base of the ovary, below the locule; (**E**) general view of flower bud of *A. vermifuga*; (**F**) enlarged view of (**E**) (rectangle), showing intercalary meristem responsible for gynophore elongation, located in the gynophore itself; (**G**) general view of flower bud of *V. macrocarpa*; (**H**) enlarged view of (**G**) (rectangle), showing intercalary meristem responsible for gynophore elongation, located in the gynophore itself; (**I**) general view of flower bud of *L. brachystachyum*; (**J**) enlarged view of (**I**) (rectangle), showing intercalary meristem (highlighted area) responsible for gynophore elongation, located in the receptacle, in the regions below the gynophore and between it and the hypanthium. Abbreviations: Gp = Gynophore, H = Hypanthium, Lo = Locule, O = Ovary, Ov = Ovule. Scale bars: (**H**,**J**) = 50 μm; (**B**,**D**,**F**) = 100 μm; (**A**,**C**) = 200 μm; (**E**,**I**) = 400 μm; G = 500 μm.

The intercalary meristem responsible for elongating the gynophore forms in the region located below the carpel suture in *A. vermifuga* (Figure 8E,F) and *V. macrocarpa* (Figure 8G,H). In *L. brachystachyum*, the intercalary meristem was observed in the center of the receptacle below the gynoecium and between the gynoecium and the hypanthium (Figure 8I,J).

In *A. hypogaea*, the intercalary meristem, responsible for the formation of the hypanthium, is seen below the insertion of the sepals, petals, and stamens (Figure 8A,B). The intercalary meristem responsible for the elongation of the gynophore after fertilization of the ovary is also already present at the base of the ovary (Figure 8C,D).

**Figure 9:**
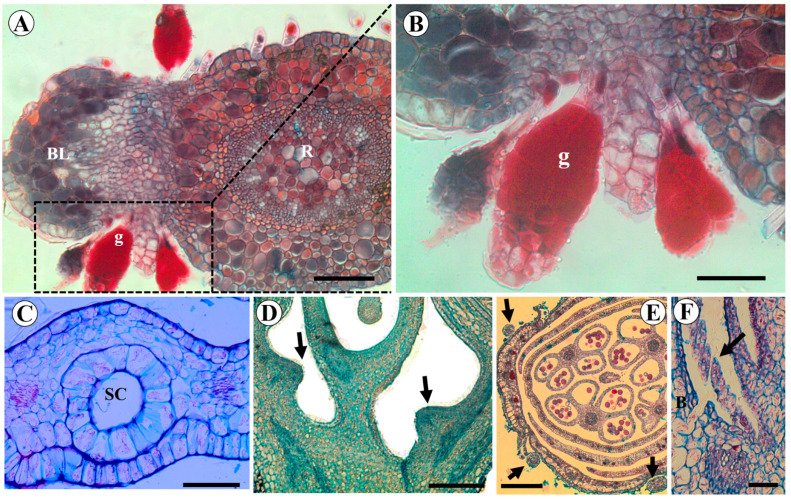
Anatomy of the secretory structures of *Leptolobium brachystachium* (**A**,**B**), *Crotalaria paulina* (**C**,**D**) and *Arachis hypogaea* (**E**,**F**). (**A**) Colleters-like glands in the axil of the bracteoles of *L. brachystachium*; (**B**) enlarged view of the colleters-like glands of (**A**) (square); (**C**) secretory cavities in wing petals of *C. paulina*; (**D**) hypanthial nectary (arrows) in *C. paulina*; (**E**) glands around the calyx (arrows) in *A. hypogaea*; (**F**) glands in the axils of the bracts (arrow) of *A. hypogaea*. Abbreviations: B = Bract, BL = bracteole, SC = secretory cavity, g = glands, R = receptacle. Scale bars: (**B**) = 50 μm; (**A**,**C**,**F**) = 100 μm; E = 200 μm; D = 400 μm.

There are colleter-like glands in the axils of the bracteoles of *L. brachystachyum* (Figure 9A,B). In *C. paulina*, there are large secretory cavities on the base of the wing petals, where the edges of these petals are wavy (Figure 9C). There is also a dilated region in the wall of the hypanthium around the base of the ovary, which probably corresponds to a hypanthial nectary (Figure 9D). In *A. hypogaea*, some trichomes are present on the epidermis of the sepals and in the axil of the bracts (Figure 9E,F).

### 2.4. Vascularization Pattern

We describe four patterns of vascularization for the studied species.

(1) Andira–Vatairea

**Figure 10:**
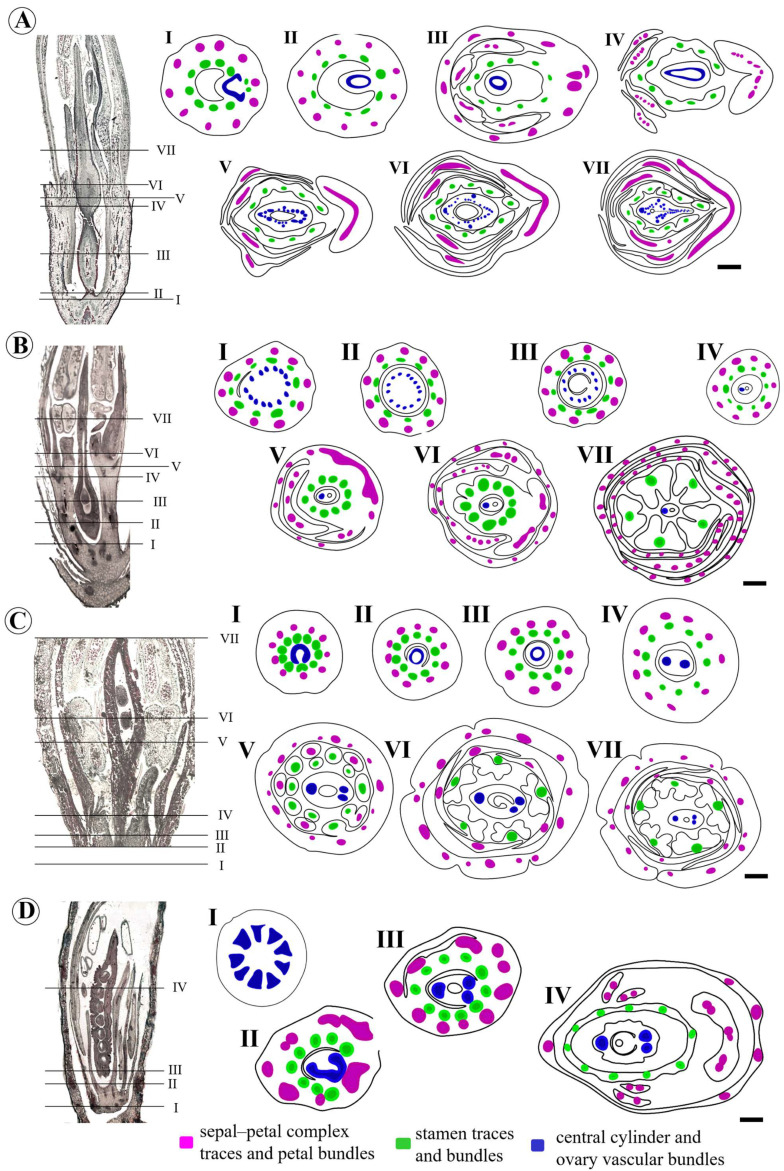
Schematic representation of the vascularization of floral bud. (**A**) Longitudinal section of flower bud of *V. macrocarpa* indicating the levels of the cross-sections represented by the diagrams (**I**–**VII**). The stele does not end in the receptacle (**I**,**II**). It continues in the gynophore region to the base of the ovary (**III**,**IV**), from which the carpel traces depart, which in turn, are subdivided into several vascular bundles that irrigate the carpel (**V**–**VII**); (**B**) longitudinal section of flower bud of *A. hypogaea* indicating the levels of the cross-sections represented by the diagrams (**I**–**VII**). The bundles of the vascular cylinder of the receptacle (**I**) continue in the region of the ovary (**II**,**III**) and decrease, leaving only one bundle in the style (**IV**–**VII**); (**C**) longitudinal section of flower bud of *L. brachystachyum* indicating the levels of the cross-sections represented by the diagrams (**I**–**VII**). The stele does not end in the receptacle (**I**,**II**). It continues in the gynophore region (**III**,**IV**), from which two carpel traces depart (**IV**), which in turn, form the three vascular bundles that irrigate the carpel (**V**–**VII**); (**D**) longitudinal section of flower bud of *Z. latifolia* indicating the levels of the cross-sections represented by the diagrams (**I**–**IV**). *Z. latifolia* does not have a gynophore, so the carpel traces depart from the receptacle stele (**I**,**II**), forming the three vascular bundles that irrigate the carpel (**III**,**IV**). Scale bar: (**B**,**C**) = 200 µm, (**D**) = 300 µm, (**A**) = 400 µm.

*Andira vermifuga* and *V. macrocarpa* have similar vascularization patterns. In the receptacle, after the departure of the vascular traces of the stamens from the central cylinder (Figure 10A(I,II)), the stele continues in the gynophore (Figure 10A(III)) until the separation of the carpel traces in the region below the carpel suture (Figure 10A(IV,V)). Starting from the locular region, the vascular bundles of the stele divide into a dorsal vascular bundle, two ventral bundles, and several small lateral bundles around the locule and others close to the carpel suture (Figure 10A(V–VII)).

(2) Arachis

After the departure of the vascular traces of the perianth and stamens into the receptacle (Figure 10B(I)), the vascular cylinder, composed of 14 vascular bundles, continues to the base of the ovary (Figure 10B(II)). From the locular region, this number reduces to 12 vascular bundles that surround the locule (Figure 10B(III)). And finally, only one vascular bundle remains in the style (Figure 10B(IV–VII)).

(3) Leptolobium

In the receptacle, the vascular traces of the perianth and stamens depart from the central cylinder (Figure 10C(I)). After the departure of the stamen traces, the stele continues in the gynophore (Figure 10C(II,III)) until the separation of the two carpel traces (one ventral and the other dorsal) in the region below the carpel suture (Figure 10C(IV)). Above the hypanthium, in the region of the ovary, the ventral trace divides into two vascular bundles (Figure 10C(V–VII)).

(4) Crotalaria–Zornia

*Crotalaria paulina* and *Z. latifolia* exhibit similar vascularization patterns. The receptacle has about 14 vascular bundles surrounding the central medulla in *Crotalaria* and 9 vascular bundles in *Zornia* (Figure 10D(I)). The divergence of the carpel traces in both species occurs in the receptacle, after the perianth and stamen traces leave (Figure 10D(II)). The stele bundles come together in three traces (two ventral and one dorsal) that are observed from the base of the carpel to the style of *C. paulina*. In *Z. latifolia*, two traces are first formed at the base of the carpel, and in the locular region, the ventral trace is divided into two (Figure 10D(III,IV)).

We summarize in Table 2 the data obtained here regarding the nature and origin of the gynophore in the clades investigated. We emphasize that the evolutionary interpretations proposed represent preliminary hypotheses, limited by the available taxonomic sample. In species with a basal pedestal, this structure contains a stele, indicating an anatomical organization consistent with an extension of the floral axis, as does the gynophore. Only the species representing the Leptolobieae clade exhibits a gynophore that originates from the receptacle; in all other species, the gynophores arise from the basal pedestal. By contrast, in species lacking a basal pedestal, the divergence of the carpel traces occurs at the level of the receptacle and, consequently, the three carpel vascular bundles are already visible at the base of the ovary. These species do not develop a gynophore.

## 3. Discussion

### 3.1. Comparison of Morphological Characters Between the Investigated Species and Other Meso-Papilionoid

Papilionoid flowers are generally zygomorphic, gathered in racemes or panicles, whose buds are subtended by a bract and two bracteoles, and display a pentamerous bauplan with 21 floral organs: 5 sepals, 5 petals, 2 whorls with 5 stamens each, and a plicated carpel unilocular. The gamosepalous calyx, usually five-lobed, the papilionaceous corolla, and the staminal filaments fused into a tube are usually elevated by the hypanthium. Although this is the general pattern observed, a variety of floral morphologies are found in what is the largest subfamily of Fabaceae, with approximately 14,000 species [12]. The Meso-Papilionoideae clade sensu Wojciechowski [1] or 50 kb inversion clade comprises 98% of these species. The flowers investigated in this work represent a tiny fraction of the morphological diversity of this clade. To provide greater robustness to the results, the morphological characteristics of the species investigated here were compared with other species from their respective clades. The results are discussed from an evolutionary perspective.

The dalbergioid species *A. hypogaea* and *Z. latifolia* differ from the other papilionoids, especially in the type of inflorescence, which is spike-like. This type of racemose inflorescence is less frequent in papilionoids [12], but is also found in *Stylosanthes* Sw. [27], a sister genus of *Arachis*. *Zornia* is also distinguished from other papilionoids, including other genera of the Adesmia clade, by having paired peltate bracteoles protecting each flower [11]. Bracteoles of papilionoides are mostly small [12], rarely conspicuous, and surround the bud and even part of the fruit, as in *Zornia*. This genus shares with some species of the Adesmia clade the absence of bracts [28].

The bilabiate calyx is typical of the Genisteae group [29], such as *Genista tinctoria* L. and *Lupinus affinis* J.Agardh [30], but also occurs in *C. paulina* in this study and in other species of Crotalaria L. [8]. This type of calyx has also been reported in other genistoid species, such as *Vuralia turcica* (Kit Tan, Vural & Küçük.) Uysal & Ertuğrul [31], and in dalbergioid species, such as *Aeschynomene sensitiva* Sw. [14], *Discolobium pulchellum* Benth [32], and *A. hypogaea* in this study. The calyx offers support and direction to the petals during the pollination process. In the bilabiate type, the upper lip supports the standard petal and prevents it from bending backwards beyond a certain point, while the lower lip supports the keel and regulates its downward movement [8].

A typical papilionaceous corolla has an outermost median (standard) adaxial petal, and pairs of differentiated lateral (wings) and abaxial (keel) petals. The keel petals are usually fused and surround the staminal tube and carpel [33]. However, several species exhibit a corolla that is either nearly papilionaceous or non-papilionaceous. The genus *Lutzelburgia* is an example of a nearly papilionaceous corolla, as its flowers have nearly free stamens and indistinct, free lower petals [34]. The independent evolution of non-papilionaceous floral architecture has been so recurrent among papilionoid legumes [3,4,35]. The Leptobieae clade is a great example of this. The genera *Leptolobium* Vogel and *Guianodendron* Sch.Rodr. & A.M.G.Azevedo have radially symmetrical flowers, while *Bowdichia* Kunth, *Diplotropis* Benth., and *Staminodianthus* D.B.O.S.Cardoso, H.C.Lima & L.P.Queiroz have nearly papilionaceous flowers [6,7,35]. Other examples include the radially symmetrical flowers of the dalbergioid genera *Riedeliella* Harms [32] and *Acosmium* Schott [36], the genistoid genus *Cadia* Forssk. [37], and most genera of the Exostyles clade [38].

The lamellate or lunate sculpturing or “bee ladders” of the wing petals is common in papilionoid flowers and is usually referred to as an adaptation to bee pollination [39]. Among the species investigated in this study, only *L. brachystachyum* does not present these sculptures. Indeed, the floral architecture and the absence of lamellate or lunate sculptures in *L. brachystachyium* may be related to its pollination syndrome, since this species is probably pollinated by wasps and not by bees, like its sister species *L. dasycarpum* Vogel and *L. elegans* Vogel [40]. The absence of lamellar sculpture is a synapomorphy of the Leptolobieae clade [35].

In *C. paulina*, a pair of callosities was observed at the base of the standard blade. The presence of callosities in the standard has systematic value to the tribe Crotalarieae. This character separates the genus *Crotalaria*, which invariably has paired callosities, from the sister genus *Bolusia* Benth., which has a single callosity. In *Crotalaria*, the callosities act as a lever for the pollinator to push against, lifting the standard petal up to reach the nectar through the nectar window [8].

The species investigated here exhibit a variety of staminal filament fusion types that reflect the diversity of floral architectures exhibited by Papilionoideae. Most papilionoid flowers have stamens united for more than half the length of the filament [4,12]. Some genera have filaments slightly fused at the base, such as *Luetzelburgia* [34]. Most species with actinomorphic flowers, such as *Leptolobium*, have free stamens, but there are exceptions, such as the genus *Riedeliella*, which has stamens fused basally into a miniature tube [32]. *Crotalaria* and *Vatairea* present a kind of transitional state between monadelphous and diadelphous androecium, in which the vexillary stamen is fused to the tube on only one side, leaving an opening in the base of the staminal tube, called “pseudo-fenestrae” by Klitgaard [41] or “nectar window” by Prenner [42]. The occurrence of a nectar window has likewise been reported in another species of *Crotalaria*, namely *C. pallida* Aiton [43]. However, unlike *C. paulina*, which keeps the sheath completely open adaxially (but overlaid), in *C. pallida* Blanco, the sheath is closed distally due to the fusion of the margins of the filament sheath, and only a single nectar window remains open basally. A similar condition to that of *C. pallida* was reported in species of Dipterygeae and Dalbergieae clades [41,44]. Regarding the diadelphous androecium observed in *A. vermifuga*, it is assumed that it increases pollination efficiency or is the result of adaptation to specific pollinators since the fusion at the base of the filamentous tube, leaving two narrow slits on the adaxial surface, allows the entry of pollinators [45].

The dimorphic anthers are considered typical structures of species belonging to the Genisteae clade [46]. This condition was observed in this study in the genistoid species, *C. paulina*, and also in the dalbergoid species, *A. hypogaea* and *Z. latifolia*. It was also documented in another dalbergoid species, *Ctenodon falcatus* (Poir.) D.B.O.S.Cardoso, P.L.R.Moraes & H.C.Lima [14]. The anther dimorphism appears to be directly linked to the degree of keel tip rostration and is therefore strongly developed in the *Crotalaria* species. The elongation of the shorter filaments of dorsifixed anthers after anthesis helps to prevent initial self-pollination. The cristate stigma of *C. paulina* and *Z. latifolia* may also be an adaptation to pollination. In their turgid state, the trichomes probably act to isolate the pollen from the stigma during anthesis to prevent initial autogamy. If pollinators do not visit the flower and crosspollination fails, delayed selfing occurs when the dorsifixed anthers elongate [8].

Regarding the presence of secretory structures, all members of the Adesmia clade share the presence of glands in some part of the plant, such as leaflets, stipules, and bracteoles [47]. *Zornia* shares secretory cavities in leaflets with the most closely related genera *Amicia* and *Poiretia* [48]. In the axils of the bracts and bracteoles and at the base of the floral bud of *L. brachystachyum*, colleter-like glands are observed. These glands are shared by all members of the Leptolobieae clade, such as *Bowdichia virgilioides* Kunth [26] and *Leptolobium elegans* Vogel [49], but are not exclusive to this clade. Similar glands have also been reported in members of the Pterocarpus clade, such as *A. hypogaea* in this study, *Pterocarpus rotundifolius* (Sond.) Druce, *Platymiscium floribundum* [41], and *Riedeliella graciliflora* Harms [32]. These glands have also been documented in other clades, such as *Holocalyx balansae* Micheli and *Zollernia ilicifolia* (Brongn.) Vogel (Exoslyleae clade) [50], and *Gliricidia sepium* (Jacq.) Kunth (Robinioid clade) [49]. It is likely that these glands have a wide distribution among papilionoids and that future studies of other taxa will reveal this.

Colleters are multicellular secretory structures attached to the stipule, petiole, lamina, bract, bracteole, calyx, and corolla, whose secretion probably protects developing meristems against desiccation [51] and pathogen attack [52]. The glands observed in *Bowdichia* and *Leptolobium* anatomically resemble the trichomatous colleters observed on the vegetative buds of Anacardiaceae species, which correspond to multicellular and multiseriate glandular trichomes, ovoid or club-shaped [49,53].

In core eudicots, the most important nectary innovation is the advent of the intrastaminal nectariferous disc [54]. None of the species investigated here have a nectariferous disc. However, in *C. paulina*, the nectar is likely released through modified stomata situated in a dilated region of the hypanthium wall that surrounds the base of the ovary, below the point of insertion of the filaments. This type of structured nectary has also been reported in the Dipterygeae, Genistoid, Dalbergioid, and Mirbelioid clades [17,31,41,42,44].

Morphological comparisons between species of the same clade show that genetically close individuals can exhibit diverse floral architectures. As an example, we can mention the clades Leptolobieae, Andira, and Dalbergioid, marked by an independent evolution of radially symmetrical flowers in some species [6]. On the other hand, individuals from different clades may present parallel evolution, showing similar floral architectures, which may be the result of a reexpression of latent genetic mechanisms [55]. The floral morphological diversity exhibited by meso-papilionoids demonstrates that, in addition to genetic systems, the evolution of flowers is strongly influenced by other factors [56]. Epigenetic and hormonal influences can alter gene expression in the apical meristems [57,58]. The selection mediated by pollinators also exhibits a strong influence on floral evolution, especially in flowers with specialized pollination systems, such as monosymmetric flowers [56]. Examples of this are the bilabiate calyx, dimorphic anthers, nectar windows, and lamellate-lunate sculpturing observed in disparate clades in this study.

### 3.2. Ovary Development in Meso-Papilionoideae

The morphological diversity of flowers results from subtle developmental changes, which can be triggered by various causes that affect the position, shape, and size of organs [56]. Evolutionary changes that occur early in ontogeny can be important drivers of morphological diversification [55]. According to Tucker’s hierarchical-significance hypothesis [59], generalized character states are expressed in the early stages of ontogeny, while specialized character states are expressed late. In this way, the first expressed states (floral symmetry, number, and position of organs) are generally stable and characterize suprageneric levels of hierarchy, while specialized states, such as fusion between organs, generally characterize genera or species. In fact, the general pattern of initiation of floral organs in Papilionoideae is unidirectional, starting on the abaxial side, and specializations, such as stamen fusion, hypanthium, and gynophore elongation, occur in the late stages of development [60].

The ontogeny of the carpel in Fabaceae is well conserved among the species of this family. Early carpel initiation is a pattern among legumes, occurring together with the petals or stamens [60]. In *Z. latifolia* and *A. hypogaea*, the initiation of the carpel together with the petals may not be an advance in the initiation of the carpel but rather a delay in the initiation of the petals because the formation of common primordia occurs in these species. In common stamen-petal primordia, petal development is often delayed, only reaching a larger size before anthesis [61]. In addition to the Dalbergioid s.l. clade [14], the occurrence of common primordia in Papilionoideae has been documented in the Hedysaroid, Astragalean, Wisterieae, and Vicioid clades (IRLC clade) [62,63,64,65,66].

In the intermediate stages, the carpel margins expand to form the carpel cleft. Subsequently, the margins are completely fused, forming an adaxial carpel suture. In some species, the carpel suture extends to the base of the carpel, while in others it does not. In the latter case, there is a region below the carpel suture, which has already been observed and highlighted in some studies [22,23,24,25,26,31]. This region is called the “basal pedestal” by Tucker [25], which suggests that this region is a precursor to the gynophore [22].

The emergence of additional structures during late floral development is also a frequent factor in floral evolution that leads to a progressive change in morphologies [56]. The development of a hypanthium or gynophore through the expansion of the floral meristem provides space for the addition of new characteristics to the flower [58]. In all papilionoid taxa that possess a hypanthium and gynophore, elongation of these structures occurs in late stages of development [60,67]. Both the gynophore and the hypanthium form from the expansion of the floral meristem [58] through the activity of intercalary meristems [18,68]. In the formation of the hypanthium, the receptacle increases in height, and the calyx, corolla, and stamens are raised [19].

### 3.3. Origin and Nature of the Gynophore

The gynophore is a stalk that elevates the gynoecium of some angiosperms. It is formed at the base of the gynoecium from the elongation of the internode between the androecium and gynoecium [19]. Gynophores are concentrated in malvids, and Fabales in fabids [69]. In Fabaceae, it is predominantly called stipe [12]. The function of the gynophore may be related to pollination, as it elevates the gynoecium above the base of the flower, providing space for the nectary and/or nectar within the nectar chamber [70]. In addition to this function, an ecological study showed that the elongated gynophore of the flower of *Breynia vitis-idaea* (Burm.f.) C.E.C.Fisch. (Phyllanthaceae) may have evolved as a defense to limit the costs of mutualism [71].

Etymologically, the term gynophore refers to a stem distinct from the gynoecium (bearer of the gynoecium) [72], and, as such, it has a stem-like nature, similar to the floral axis, and its origin is receptacular, as it consists of an extension of the receptacle [73]. According to Periasamy and Sampoornam [74], stalked gynoecia are not rare in angiosperms, but the question is whether all such stalks can be called gynophores. In *Bowdichia. virgilioides* the gynophore is receptacular because the intercalary growth that leads to elongation of the gynophore occurs in the receptacle region [26]. In the present study, we found that the gynophore also has a receptacular origin in *L. brachystachyum*, which belongs to the same clade as *B. virgilioides* (Leptolobieae clade). In contrast, the intercalary meristem is located below the gynoecium in *A. hypogaea*, *A. vermifuga,* and *V. macrocarpa*.

The presence of the intercalary meristem at the base of the ovary of *A. hypogaea* has been well documented and comprises small cells arranged in vertical files (Figure 8 in [75]). The gynophore of the *Arachis* species is peculiar because it is related to the geocarpic habit. Ovary development after pollination stimulates the intercalary meristem, causing gynophore extension [75]. It has even been suggested that the name gynophore would be inappropriate for this structure in *Arachis* since its origin is found at the base of the carpel [20]. However, what is intriguing about this structure is that, despite its origin (receptacle or ovary base), its nature is stem-like in all species investigated here. Our anatomical studies show that this region presents a ring of vascular bundles around the center (medulla) in an arrangement similar to the typical anatomy of a eudicot stem, configuring an eustele. Therefore, the stem-like nature is present in both the gynophores of the carpel base and the receptacular origin. Our explanation for this condition is that the base of the carpel of species that have a gynophore consists of an internode, similar to the solid basal region found between the gynoecium and stamens of flowers of *Arabidopsis* and other members of the Brassicaceae [76]. In *Arabidopsis* mutants, this internode is seen below the gynoecium before the formation of the lateral crease of the gynoecium [77]. In Fabaceae, this internode is called the “basal pedestal” [25]. In an anatomical study of *Bowdichia virgilioides,* we observed that, before gynophore elongation, the divergence of carpel traces occurs at the basal pedestal, not the receptacle. After gynophore elongation, the divergence of carpel traces occurs at its apex. This indicates that the basal pedestal corresponds to an extension of the floral axis and that gynophore elongation occurs from this region [26]. In *Erythrina lysistemon* Hutch., fourteen collateral vascular bundles were counted in the center of the pedicel, and they were also present in the gynophore. Corroborating our observations, at the base of the ovary cavity of *E. lysistemon*, the gynophore bundles fuse to form three vascular traces that run to the ovarian wall and form a large dorsal bundle and two ventral bundles [78]. In *Tachigali* Aubl., it was also observed that the vascular cylinder of the gynophore is transformed into three vascular bundles in the ovary [16]. In the classic study on the morpho-anatomy of legume carpels by Leinfellner [79], schematic illustrations show that, in pedunculate carpels, the divergence of carpel traces occurs within the gynophore, as observed in *Erythrina crista-galli* L. In contrast, sessile carpels display the three carpel bundles originating at the base, since their divergence takes place in the receptacle, as seen in *Lupinus polyphyllus* Lindl.

The ovary of *A. hypogaea* appears sessile, but there is a very short region below the carpel suture where the inactive intercalary meristem is located. Jacobs [21] called this region the “small ovarian stalk”. Our studies showed that this region has similar anatomy to the gynophore. The basal pedestal is not present in species that do not have a gynophore, such *as Duparquetia orchidacea* Baill. [80], *Abrus precatorius* L. [81], *Z. latifolia,* and *C. paulina*, because in these species, the carpel cleft closure extends to the base of the carpel. In *Z. latifolia* and *C. paulina,* the divergence of carpel features occurs in the receptacle, and the three vascular bundles are visualized from the base of the carpel in anatomical cross-sections.

Leinfellner [79] documents cases in which the ventral fissure of the carpel extends into the gynophore, or even beyond it, reaching the floral receptacle, as in *Hallimodendron halodendron* (Pall.) Voss, renamed *Caragana halodendron* (Pall.) Dum.Cours. The study classifies the gynophores of papilionoid species as totally unifacial, unifacial at the base and bifacial apically to varying degrees, or completely bifacial, depending on the extent of the ventral fissure in the gynophore. A gynophore lacking a ventral fissure is considered unifacial because its vascular supply consists of a continuous ring of vascular bundles. When the gynophore is affected by a ventral carpel fissure, it becomes totally or partially bifacial, depending on how far the fissure extends. However, even in gynophores described as totally or partially bifacial, schematic figures show a ring of vascular bundles that is interrupted only in the ventral region. This ventrally open, horseshoe-shaped ring originates from a closed vascular ring of the floral axis and divides into dorsal and ventral bundles only at the base of the carpel itself. In all species analysed in our study, the ventral carpel cleft terminates at the apex of the gynophore and is therefore unifacial along its entire length. Further investigations into the development and vascularization of species with atypical gynophores, as described by Leinfellner, would be valuable in complementing the observations presented here.

Studies of floral vascular anatomy in Papilionoideae reveal considerable diversity in vascularization patterns, providing insights that inform updated phylogenetic and evolutionary interpretations and help elucidate the nature of floral structures within the family [82,83]. Here, species with similar external morphologies exhibited comparable vascularization patterns, despite belonging to different clades. Moreover, the vascular anatomy helped elucidate the nature of the gynophore, whose vascularization represents a continuation of the receptacle’s eustele. In her theory of carpel polymorphism, Saunders [84] interpreted the residual vascular tissue of the gynophore and gynoecium as vestiges of an ancestral floral condition, in which the vascular system supplied two whorls of pentamerous carpels. In the genus *Arachis*, this condition is interpreted as still discernible, as the vascular bundles remain organized as ten independent strands corresponding to ancestral carpels fused into a simple gynoecium. In contrast, Moore [85] argued that the extension of the gynophore eustele into the ovary wall of *Arachis* represents a condition markedly distinct from that commonly observed in other legumes. This distinctive morphological feature is therefore likely to be associated with the geocarpic habit of the genus.

Leinfellner [79] considered the gynophore as part of the carpel and analogous to the leaf petiole. However, he also acknowledges that the gynophore of the terminal carpel in legumes often exhibits an axial appearance and that the vascular ring of the gynophore frequently represents a direct continuation of the floral axis’s vascular ring, following the separation of the perianth and stamen traces. Despite these observations, the author downplays their significance, arguing that they do not indicate a stem-like nature but rather reflect a unifacial structure, which arises because a rearrangement of the vascular cylinder bundles from the floral axis in the gynophore region is unnecessary.

The distinction between terminal and lateral carpels provides an important framework for interpreting gynoecial structure and the nature of the gynophore. In taxa with a unicarpellate gynoecium occupying a terminal position, the entire floral apex is converted into a carpel, and no residue persists laterally around the base of the carpel [86,87]. In contrast, a lateral origin for the unicarpellate gynoecium of legumes has also been proposed [88]. Sinjushin [13] highlighted a protuberance, corresponding to the floral axis, observed just below the adaxial carpel cleft, during the initiation of the carpel of *Cordyla pinnata*. This same protuberance can also be observed in the development of the carpel of different species of the genus *Swartzia*, called by Tucker [89] a convex pedestal. It is important to emphasize that, in both genera, the elongation of this protuberance in the later stages of development leads to the formation of the gynophore. In several other studies, the authors observed that the carpel was situated on a type of elevation of the receptacle, whose subsequent extensions led to the formation of the gynophore [23,25,31,90]. These observations strengthen our hypotheses that the gynophore of legumes corresponds to a prolongation or persistence of the floral apex during carpel formation.

According to Endress [91], the floral organs are “appendages” or “lateral structures,” and this also applies to the carpels. But the gynoecium has a special position because it encloses the development of the floral apex. The floral apex may be completely consumed by the developing gynoecium, or a residual part is left beside the gynoecium if it is unicarpellate, or in the center of the gynoecium, especially when the gynoecium is polycarpellate [92,93]. The occurrence of both positional configurations within the Fabaceae supports the interpretation of the angiosperm carpel as a composite structure, in which axial and foliar components are differentially expressed [54]. This reinforces the importance of the gynophore as a key character for evolutionary studies of the gynoecium. Finally, we conclude that the species analysed here have two types of gynophore sensu stricto: one that originates from the basal pedestal and another that originates from the receptacle, but both have a stem-like nature. The gynophore is the result of the elongation of the basal pedestal itself (which consists of an internode) when the intercalary meristem emerges in this region. This is what happens in *A. hypogaea*, *A. vermifuga*, and *V. macrocarpa*. But the gynophore may also result from the lengthening of the internode between the gynoecium and the stamens when the intercalary meristem emerges into the receptacle. In the latter case, the elongation of the gynophore elevates the basal pedestal, as occurs in the genistoid species, *B. virgilioides* [26], and *L. brachystachyum*.

## 4. Materials and Methods

### 4.1. Plant Material

Inflorescences with flowers and floral buds at various stages of development were collected from native plants in the field, except *Arachis hypogaea*, which was obtained from cultivated specimens (Table 3). Vouchers were deposited at the herbarium of the University of Brasilia (Herbarium UB). The samples collected were preserved in 70% FAA (5 parts formalin, 5 parts acetic acid, and 90 parts 70% ethyl alcohol) for 48 h, and stored in 70% ethanol.

### 4.2. Floral Development Analysis Under Scanning Electron Microscopy

The study of floral development was carried out on the following species: *A. vermifuga*, *A. hypogaea*, *C. paulina*, *L. brachystachyum*, *L. auriculata*, and *Z. latifolia*. Samples stored in 70% ethanol were examined and dissected with the help of a stereomicroscope (EZ4 W, Leica Microsystems, Wetzlar, Germany). Following dissection, samples were dehydrated in an ethanol series 70%, 80%, 90%, and 100% for at least 12 h each step, and then critical-point dried in an evaporator (Baltec Device CPD 030, Balzers, Liechtenstein). Pieces were mounted on metal supports (stubs) with carbon tape and treated with gold in a sputter coater (EM SCD 500, Leica Microsystems, Wetzlar, Germany). The buds were studied and micrographs taken with a scanning electron microscope (equipment JSM-7001F JEOL Ltd., Akishima, Tokyo, Japan) at 15 Kv. The electron micrographs were processed using Adobe Photoshop software version 25.9.1. All steps were performed at the Microscopy and Microanalysis Laboratory of the Institute of Biological Sciences (IB) of the University of Brasilia (UnB), Brasilia, Brazil.

### 4.3. Anatomical Study

The anatomical study of the following species was carried out: *A. vermifuga*, *A. hypogaea*, *C. paulina*, *L. brachystachyum*, *V. macrocarpa*, and *Z. latifolia*. Floral buds in the late stages of development (stored in 70% ethanol) were gradually dehydrated in an ethanol series 70%, 80%, 90%, and 100% at room temperature for at least 2 h at each concentration. Subsequently, the buds were diaphanized with ethanol plus n-butyl acetate, in the proportions 3:1, 1:1, and 1:3, at room temperature for at least 2 h for each step, and finally pure n-butyl acetate (overnight). The material obtained was embedded in paraffin [94] at 60 °C for 24 h. This procedure was repeated two more times after changing the paraffin. The samples were embedded in paraffin in handmade paper boxes. After hardening the material at room temperature for 24 h, it was assembled on a wooden support and sectioned transversely and longitudinally (10–15 µm thick) in a rotary microtome (RM 2145, Leica Microsystems, Wetzlar, Germany). The serial sections were deposited on histological slides, which were deparaffinized using pure butyl acetate, butyl acetate plus ethanol (in proportions 3:1, 1:1, and 1:3), and ethanol 100%, 90%, 80%, 70%, 60%, and 50%. The sections were stained with 1% alcoholic safranin 1% for 20 min, and 1% alcoholic astra blue for 2 min [95]. The sections were subjected to an alcoholic series (60%, 70%, 80%, 90%, and 100%), ethanol plus n-butyl acetate (3:1, 1:1, and 1:3), and pure n-butyl acetate. Finally, the sections were mounted in synthetic resin [96]. The anatomical sections were observed and photographed under a light photomicroscope (BX40, Olympus Optical Co., Hachioji, Tokyo, Japan) coupled to a digital camera (SC30, Olympus Optical Co., Hachioji, Tokyo, Japan) with the scale bars at the same optical conditions. All procedures were carried out at the Botany Department of the Institute of Biological Sciences, University of Brasilia, Brasilia, Brazil. From the anatomical sections, floral vascularization schemes were created using Adobe Illustrator version 28.5 software.

The terminology and abbreviations used to describe the floral ontogeny followed Tucker [25,60,67], and the flower morphology and anatomy followed Radford et al. [97], Fahn [18], and Endress [45]. The floral diagram was based on Ronse De Craene [73], and the format of floral formulae was based on Prenner et al. [98].

## 5. Conclusions

The flowers investigated here present morphological characters that represent a tiny fraction of the great diversity of floral architectures present in papilionoid legumes. Most variations are typically homoplastic characters and are generally related to pollination syndrome. This morphological diversity was used to analyze the origin and nature of the gynophore. In species with this structure, an internode or basal pedestal forms at the beginning of carpel development and is located below the carpel suture. In some species, the intercalary meristem emerges in this region in the final stages of development, forming the gynophore. Other species have the intercalary meristem come out of the receptacle, and its extension raises the basal pedestal and makes the gynophore. Therefore, our results highlight two origins of the gynophore in papilionoid legumes and support the interpretation of the gynophore as an axial structure. Of the papilionoid clades represented here, only the Leptolobieae clade presented a gynophore of receptacular origin, while the gynophores of the Andira, Vataireoid, and Pterocarpus clades originated from the basal pedestal. The representative species of the Crotalarieae and Adesmia clades did not possess a gynophore. In these species, the basal pedestal is not formed, and the carpel suture extends to the base of the carpel. In these, the carpel traces depart from the eustele in the receptacle region, unlike species that have a gynophore, in which the carpel traces depart from the gynophore in the region just below the carpel suture. However, we emphasize that these conclusions are preliminary and represent hypotheses derived from a qualitative comparative analysis based on a limited taxonomic sample. Our interpretations aimed to establish a consistent anatomical framework to recognize the axial nature of the gynophore and distinguish its different developmental origins, providing a necessary basis for future analyses. The evolutionary scenarios proposed here will require further testing using a broader taxonomic sample, integrated with quantitative approaches and phylogenetic analyses, contributing to a more robust assessment of the evolutionary importance of the gynophore in Papilionoideae.

## Figures and Tables

**Table 1:** Comparison of developmental and morphoanatomical character states, in the seven taxa studied, including floral diagrams and formulae.

Clade	Andira	Vataireoid		Genistoid s.l.		Dalbergioid s.l.	
				Crotalarieae	Leptolobieae	Pterocarpus	Adesmia
Species	*A. vermifuga*	*L. auriculata*	*V. macrocarpa*	*C. paulina*	*L. brachystachyum*	*A. hypogaea*	*Z. latifolia*
Floral diagram	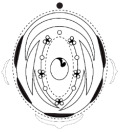	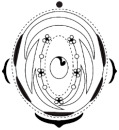	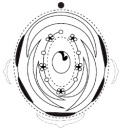	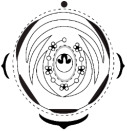	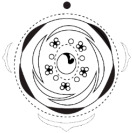	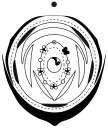	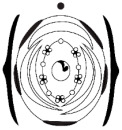
Inflorescence	Panicles	Panicle	Panicle	Raceme	Panicle	Spike	Spike
Bracts and bracteoles	Caducous	Persistent	Caducous	Persistent	Caducous Colleteres-like glands	Persistent Colleteres-like glands	No bract Peltate bracteoles Transparent glands
Calyx	Campanulate	Campanulate	Campanulate	Bilabiate	Campanulate	Bilabiate	Campanulate
Corolla	Papilionaceous	Nearly Papilionaceous	Papilionaceous	Papilionaceous	Non-papilionaceous	Papilionaceous	Papilionaceous
Floral symmetry	Zygomorphic	Zygomorphic	Zygomorphic	Zygomorphic	Actinomorphic	Zygomorphic	Zygomorphic
Lamellate sculpturing	Present	Present	Present	Present	Absent	Present	Present
Androecium	Diadelphous	Monadelphous, Connate in the base	Monadelphous, open stamen tube	Monadelphous, open stamen tube	Free	Monadelphous	Monadelphous
Anthers	Monomorphic	Monomorphic	Monomorphic	Dimorphic	Monomorphic	Dimorphic	Dimorphic
Staminodes	0	0	0	0	0	2	0
Organ loss or suppression	0	Vexillary stamen	0	0	0	0	0
Carpel initiation	Concurrently with antesepalous stamens	Concurrently with antesepalous stamens	—	Concurrently with antesepalous stamens	Concurrently with antesepalous stamens	Concurrently with petals	Concurrently with petals
Stigma	Cristate	Punctiform	Punctiform	Cristate	Punctiform	Clavate	Cristate
Ovules	5	1	1	20	3	2	9
Ovary anatomical shape	oblique and oblong	Unidentified	elliptical	ovoid	elliptical	globose	elliptical
Ovary vascularization	1 dorsal, 2 ventral and small bundles around the locule and close to the carpel suture	Unidentified	1 dorsal, 2 ventral, 2 lateral and small bundles around the locule and close to the carpel suture	1 dorsal and 2 ventral vascular bundles.	1 dorsal and 2 ventral vascular bundles	12 vascular bundles arranged around the locule	1 dorsal and 2 ventral vascular bundles.
Carpel outer epidermis	Biseriate Glabrous	Unidentified	Uniseriate Hairy	Uniseriate Glabrous	Uniseriate Hairy	Uniseriate Glabrous	Uniseriate Hairy (ventral and dorsal)
Carpel mesophyll	Heterogeneous	Unidentified	Heterogeneous	Homogeneous	Heterogeneous	Homogeneous	homogeneous
Gynophore	Long	Short	Long	Absent	Long	Long Present after pollination	Absent
Gynophore epidermis	Uniseriate Glabrous	Unidentified	Uniseriate Hairy	—	Uniseriate sparse trichomes	—	—
Hypanthium	Short	Short	Long	Very short	Long	Very long	Absent
Nectary	Unidentified	Unidentified	Unidentified	Hypanthial Modified stomata	Unidentified	Unidentified	Unidentified

Note: A dash indicates the absence of information; Floral formulae: *Andira vermifuga*: B Bt K(5)↓ C1^flag^: 2^wing^:2^keel^↓ A (5 + 4):1↓ G1↓ Vm5; *Luetzelburgia auriculata:* B Bt K(5)↓ C1^flag^: 2^wing^:2^keel^↓ A (5 + 4): 1^0^↓ G1↓ Vm1; *Vatairea macrocarpa:* B Bt K(5)↓ C1^flag^: 2^wing^:2^keel^↓ A (5 + 5) ↓ G1↓ Vm1; *Crotalaria paulina:* B Bt K(5)↓ C1^flag^: 2^wing^:(2)^keel^↓ A(5 + 5)↓ G1↓ Vm20; *Leptolobium brachystachyum:* B Bt K(5)↓ C5* A5 + 5↓*G1*↓ Vm3; *Arachis hypogaea:* B Bt K(5)↓ C1^flag^: 2^wing^:(2)^keel^↓ A(4:1^r^ + 4:1^r^) ↓ G1↓ Vm2; *Zornia latifolia:* Bt K(5)↓ C1^flag^: 2^wing^:(2)^keel^↓ A (5 + 5)↓ G1↓ Vm9; Floral diagram legend: 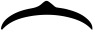 bract or bracteole; 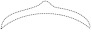 early caducous bract or bracteole; 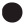 main axis relative to flower; 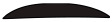 sepal; 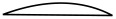 petal; 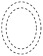 Extent of hypanthium development; 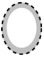 hypanthial nectary; 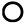 antepetalous stamen; 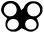 antesepalous stamen; 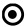 antepetalous staminode; 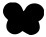 antesepalous staminode; 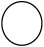 single carpel; 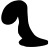 ovule; 
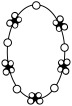
 fusion of stamens.

**Table 2:** Vascularization and origin of the gynophore in Meso-Papilionoideae.

Clade	Subclade	Basal Pedestal	Ovary Base Vascularization	Gynophore Vascularization	Gynophore Origin
Andira		present	stele	stele	Basal pedestal
Vataireoid		present	stele	stele	Basal pedestal
Genistoid s.l.	Crotalarieae	absent	1 dorsal and 2 ventral vascular bundles.	-----	-----
	Leptolobieae	present	stele	stele	Receptacle
Dalbergioid s.l.	Pterocarpus	present	stele	stele	Basal pedestal
	Adesmia	absent	1 dorsal and 2 ventral vascular bundles.	-----	-----

Legend: ----- The species examined do not possess a gynophore.

**Table 3:** Species collection data.

Species	Date	Collection Site	Voucher
*Andira vermifuga*	19 September 2020	Urucuia (Minas Gerais, Brazil)	C.G. Rodrigues 07 UB 219898
	9 March 2022	Arinos (Minas Gerais, Brazil)	C.G. Rodrigues 26 UB 219915
	13 August 2022	Arinos (Minas Gerais, Brazil)	C.G. Rodrigues 37 UB 227698
*Arachis hypogaea*	23 November 2023	Arinos (Minas Gerais, Brazil)	C.G. Rodrigues 56 UB 241621
*Crotalaria. paulina*	17 December 2020	Brasília (Distrito Federal, Brazil)	C.G. Rodrigues 16 UB 219905
*Leptolobium brachystachyum*	17 October 2022	Parque Estadual do Biribiri, Diamantina (Minas Gerais, Brazil)	C.G. Rodrigues 47 UB 227707
*Luetzelburgia auriculata*	7 August 2021	Arinos (Minas Gerais, Brazil)	C.G. Rodrigues 51 UB 241616
*Vatairea macrocarpa*	24 August 2023	Arinos (Minas Gerais, Brazil)	C.G. Rodrigues 54 UB 241619
	1 September 2023		C.G. Rodrigues 58 UB 248391
*Zornia latifolia*	4 March 2024	Arinos (Minas Gerais, Brazil)	C.G. Rodrigues 59 UB 248393
	3 April 2024		C.G. Rodrigues 60 UB 248394

Species

Date

Collection Site

Voucher

## Data Availability

The data underlying this article will be shared upon reasonable request to the corresponding author. The data are not publicly available due to the descriptive nature of the study.
